# Bioinspired Suction-Cup Architecture Modulates Hydration and Pomegranate-Extract Release from NVP/HEMA/CMC Hydrogels

**DOI:** 10.3390/gels12100860

**Published:** 2026-09-24

**Authors:** Nadda Chiaoprakobkij, Wasipim Chansiriwat, Wisanee Wisanwattana, Neti Waranuch, Thanyaporn Pinthong, Sukunya Ross, Jarupa Viyoch, Gareth Ross

**Affiliations:** 1 Biopolymer Group, Center of Excellence in Biomaterials, Department of Chemistry, Faculty of Science, Naresuan University, Phitsanulok 65000, Thailandthanyaporn.pi@nu.ac.th (T.P.); sukunyaj@nu.ac.th (S.R.); 2 Cosmetics and Natural Products Research Center (CosNat), Faculty of Pharmaceutical Sciences, Naresuan University, Phitsanulok 65000, Thailand; wisanee.wi@nu.ac.th (W.W.); netiw@nu.ac.th (N.W.); 3 Department of Pharmaceutical Technology, Center of Excellence for Innovation in Chemistry, Faculty of Pharmaceutical Sciences, Naresuan University, Phitsanulok 65000, Thailand

**Keywords:** photopolymerized hydrogel, suction-cup topography, pomegranate extract

## Abstract

Surface architecture may tune hydrogel transport without changing formulation. Flat and suction-cup N-vinylpyrrolidone/2-hydroxyethyl methacrylate/carboxymethyl cellulose (NVP/HEMA/CMC) hydrogels prepared from the same nominal formulation were compared for hydration, equilibrium water content, pomegranate-extract uptake, and 60 min release. Suction-cup hydrogels showed greater short-term swelling (38.7 ± 0.7%) than flat hydrogels (31.4 ± 0.6%), a 23.1% increase, while equilibrium water contents remained similar. Apparent extract uptake, estimated from loading-solution depletion, was 20.45 and 15.74 mg/g hydrated hydrogel for suction-cup and flat formats, respectively. After 60 min, cumulative apparent pomegranate-extract-equivalent release was 6.976 ± 0.681 and 4.208 ± 0.354 mg, respectively, representing a 65.8% increase. These values corresponded to approximately 17.6% and 15.2% of estimated initial loadings. Indirect-extract testing showed minimum viabilities of 86.24% for HaCaT cells and 71.01% for human dermal fibroblasts, exceeding the 70% ISO 10993-5 criterion. Overall, suction-cup architecture increased early swelling, estimated uptake, and cumulative release without altering formulation. Although the individual contributions of surface area, local thickness, liquid retention, and diffusion paths could not be separated, the results demonstrate that architectural modification can enhance early hydration and extract uptake and release while preserving the hydrogel formulation.

## 1. Introduction

Hydrogels are crosslinked hydrophilic polymer networks that absorb water while retaining a coherent three-dimensional form. Their high water content, adjustable mechanical properties, and capacity to incorporate soluble compounds support their use in drug delivery, wound-contact materials, and tissue engineering [1,2,3]. Hydrogel performance is commonly adjusted through monomer selection, crosslink density, or network chemistry. The macroscopic design of a hydrogel provides another variable, as the same polymer formulation can be prepared as a bulk gel, thin film [4], coating, contact lens, porous scaffold [5], microgel, or nanogel [6]. These different forms alter the exposed area, local thickness, and interfacial geometry, thereby affecting liquid access and diffusion [7].

Architecture can influence transient transport without necessarily changing the equilibrium water fraction of the polymer network. An increase in accessible interface may facilitate exchange with the surrounding liquid, whereas local thickness changes the distance over which water and dissolved compounds must travel. Concave features can also retain external liquid, which must be distinguished from water absorbed into the network. These effects depend on the complete geometry and test conditions; increasing nominal surface area alone does not establish faster hydration or release. Photopolymerization provides a practical route to investigate such structures because a liquid precursor can be cast against a patterned mold and crosslinked while retaining the imposed topography [8,9]. Bioinspired suction structures have been used to improve wet interfacial contact and adhesion [10,11,12]. Cupping-based needle-free delivery has also used negative pressure to deform skin and modify the stratum corneum [13], while artificial octopus-limb-like patches have combined wet adhesion with cupping-driven transdermal delivery [14]. Other systems couple suction structures to additional delivery components. Zhu et al. used cups to retain a microneedle patch on wet tissue [15], and Li et al. incorporated a micro-suction-cup array into a multifunctional wound patch with photothermally controlled adhesion and release [16]. These studies establish roles for suction structures in attachment and integrated delivery systems but do not isolate transport from a formulation-matched flat-versus-suction-cup hydrogel comparison. Surface patterning has separately been investigated in thermoresponsive PNIPAAm/graphene oxide hydrogel films [17]. That work supports architecture as a transport variable but concerns a different material and pattern. The unresolved question addressed here is whether fabricating the same nominal precursor formulation as flat and suction-cup hydrogels produces different short-term hydration and apparent extract-transfer responses.

The present hydrogel comprised N-vinylpyrrolidone (NVP) and 2-hydroxyethyl methacrylate (HEMA), polyethylene glycol diacrylate (PEGDA) as the crosslinker, and carboxymethyl cellulose (CMC) as a hydrophilic polysaccharide component. Related photopolymerized networks combining NVP/HEMA with chitosan have been evaluated for swelling, drug release, and HaCaT cytocompatibility [18], while poly(N-vinylpyrrolidone)/CMC hydrogels have demonstrated composition-dependent swelling and release [19]. These studies provide material context; the principal contribution here is the comparison of fabricated architectures at a fixed nominal precursor composition. The same precursor recipe does not, by itself, establish identical conversion or effective crosslink density throughout geometrically different specimens.

Accordingly, this study compared flat and suction-cup NVP/HEMA/CMC hydrogels in terms of nominal external surface area per dry mass, short-term mass uptake, EWC, and apparent pomegranate-extract transfer. Pomegranate peel extract was used as a multicomponent model cargo containing UV-active phenolic constituents, including ellagitannins [20]. Whole-extract UV measurements were interpreted as extract-equivalent responses rather than compound-specific concentrations. Mechanical and interfacial measurements provided supporting characterization, and indirect testing with HaCaT keratinocytes and human dermal fibroblasts screened the cytocompatibility of the selected extract-containing hydrogel. The comparison concerns the combined fabricated format; it does not independently isolate surface area, thickness, suction pressure, or a release mechanism.

## 2. Results and Discussion

The performance of hydrogel systems is governed by both polymer-network chemistry and macroscopic architecture. In this study, flat and suction-cup NVP/HEMA/CMC hydrogels were prepared from the same precursor formulation, allowing the influence of the fabricated geometry to be examined without changing the nominal chemical composition. The two hydrogel formats were compared in terms of fabrication, hydration and swelling, and pomegranate-extract uptake and release. Indirect cytocompatibility testing was additionally used to assess the biological response to extracts from the bare and pomegranate-extract-loaded hydrogels.

### 2.1. Fabrication of Flat and Suction-Cup Hydrogels

Replica molding was used to transfer the digitally designed suction-cup geometry into the photocrosslinked hydrogel. Flat hydrogels were cast in a planar mold, whereas the structured hydrogels were prepared using a silicone female mold from a 3D-printed male master. For the structured format, a parametric three-dimensional model was converted into a sliced print file and used to produce the male master shown in Figure 1. Following photopolymerization and demolding, the dry hydrogel retained the periodic array of suction-cup geometry. This confirmed that the designed architecture could be reproduced through the successive printing, molding, and hydrogel-fabrication stages. The nominal cup geometry comprised a height of 1.0 mm, an upper outer diameter of 4.0 mm, a lower outer diameter of 2.5 mm, and a wall thickness of 0.4 mm. Adjacent cups were separated by a center-to-center distance of 5.0 mm. These values were taken from the parametric design. Both hydrogel formats were prepared from the same precursor formulation, so their nominal polymer composition was unchanged. Hydration was then compared between the flat and suction-cup samples to determine how the change in fabricated format, including surface architecture, exposed area, and local thickness, affected water uptake.

### 2.2. Hydration Behavior and Dimensional Response of Flat and Suction-Cup Hydrogels

The suction-cup specimens showed a greater percentage mass increase than the flat specimens during the 120 min experiment (Figure 2a). At 120 min, the mass increases were 38.69 ± 0.75% and 31.44 ± 0.60%, respectively (*n* = 3, individual specimens), giving a 23.1% higher endpoint mean for the suction-cup format. This is a comparison of mass uptake relative to the initial specimen state, not a 23.1% increase in equilibrium water content. The reported EWC values were 51.00 ± 2.29% for the flat hydrogel and 51.83 ± 0.35% for the suction-cup hydrogel (Figure 2b; *n* = 3, individual specimens). EWC is the water fraction of the equilibrium hydrated specimen, whereas the uptake curve describes the change in mass relative to W_0_. The similar EWC values indicate comparable water fractions under the test conditions described in Section 4.4, although they do not necessarily imply identical polymer network structures. The initial water contact angle was 73.3° for the flat hydrogel and 86.4° for the suction-cup hydrogel (Figure 2b). However, for the suction-cup hydrogel, the measured contact angle may also be influenced by the position of the droplet relative to the cup rims and cavities. The result should therefore be interpreted as the apparent wettability of the fabricated surface rather than a difference in the intrinsic chemistry of the NVP/HEMA/CMC network.

For the nominal geometric comparison, the dry masses were 3.710 ± 0.210 g for the flat format and 3.828 ± 0.067 g for the suction-cup format. External surface area calculated from nominal dimensions and normalized by dry mass was 9.78 ± 0.55 and 11.86 ± 0.21 cm^2^ g^−1^, respectively. The resulting 21.3% increase in surface area per unit dry mass is close to the reported 23.1% increase in mass uptake at 120 min. We therefore propose that the increased surface area of the suction-cup format was the primary contributor to its higher short-term hydration, although its contribution was not independently quantified. Differences in local thickness and diffusion paths may also have influenced water uptake, while water retained within the cup cavities during weighing may have contributed to the measured mass uptake.

### 2.3. Mechanical Response of Flat and Suction-Cup Hydrogels

The tensile response of the flat and suction-cup hydrogels is shown in Figure 3b. The flat hydrogel had a mean tensile strength of 88.7 ± 5.3 kPa, compared with 78.8 ± 7.5 kPa for the suction-cup hydrogel (*n* = 5). Although the stress–strain profiles overlapped, the suction-cup specimens generally showed a lower stress response and extended further before failure. The suction-cup format was therefore more compliant, with a modest reduction in tensile strength relative to the flat hydrogel. Since both hydrogels were prepared from the same precursor formulation, this difference is attributed primarily to the fabricated architecture. The curved cup walls and changes in local cross-sectional area might allow greater deformation but may also introduce regions of stress concentration. For a hydrogel intended to contact non-planar surfaces, greater deformability may be beneficial because it allows the material to follow the surface curvature and may help maintain contact across the patterned region. Flexible hydrogel patches have similarly been shown to conform to curved and tubular surfaces [12,21].

Lap-shear testing (setup shown in Figure 3a) applies a load parallel to the contacted interface and is commonly used to assess the resistance of an adhesive joint to interfacial sliding, including hydrogel–tissue interfaces and tissue adhesives [21,22]. This loading mode is relevant to a skin-contacting patch because lateral movement of the skin could cause the material to slide or detach after application. Nevertheless, the present experiment should be regarded as an interfacial shear screening test rather than a direct measurement of adhesion to skin. Skin adhesion would also depend on surface roughness, moisture, preload, contact time, curvature, and the resistance of the material to peeling or normal detachment. Lap-shear testing results gave a shear strength of 2.87 ± 0.47 kPa for the flat hydrogel and 0.44 ± 0.20 kPa for the suction-cup hydrogel (Figure 3c). This comparison does not account for the large difference in contact area between the two formats. The flat hydrogel contacted the substrate over the full nominal area of 6.25 cm^2^, whereas the suction-cup hydrogel contacted the surface through the annular rims of nine cups, giving an estimated effective contact area of 0.63 cm^2^.

After normalization by the nominal contact area, the apparent shear strength was 0.459 ± 0.075 kPa cm^−2^ for the flat hydrogel and 0.694 ± 0.136 kPa cm^−2^ for the suction-cup hydrogel (Figure 3d). The area-normalized response of the suction-cup hydrogel was therefore 51.2% higher than that of the flat hydrogel. Although the flat specimen produced a higher overall shear strength, the suction-cup architecture provided greater shear resistance relative to the area in contact with the substrate. This indicates that the cup rims used the available contact area more efficiently during shear loading. The normalized comparison depends on the estimated annular contact area and the number of cups engaged with the substrate.

### 2.4. Pomegranate-Extract Loading and Release from Flat and Suction-Cup Hydrogels

Both hydrogel formats developed a uniform amber coloration after immersion in the pomegranate extract, while the suction-cup array remained clearly defined (Figure 4a). The color change is consistent with the uptake of extract components into the hydrated polymer network. However, the photographs provide only qualitative evidence and cannot establish either the internal distribution or the amount of extract retained by each hydrogel format. Release was monitored by measuring absorbance at 290 nm. Pomegranate peel extract contains several UV-active phenolic constituents, including ellagitannins and their related products [20]. Since these compounds can contribute differently to the measured absorbance, the calibration prepared from the complete extract reports pomegranate-extract-equivalent concentration rather than the concentration of punicalagin or any other individual compound. The results therefore represent the combined UV response of the extract released from the hydrogel.

Initial pomegranate-extract uptake was estimated from the decrease in extract concentration in the loading solution. After loading, the mean concentration decreased from 2.00% to 1.77% for the flat hydrogels and 1.67% for the suction-cup hydrogels. For a loading-solution volume of 12 mL, these changes corresponded to apparent uptake amounts of 27.6 and 39.6 mg, respectively. Dividing these amounts by the mean hydrated specimen masses of 1.754 and 1.937 g gave uptake values of 15.74 and 20.45 mg/g hydrated hydrogel, respectively. The mass-normalized uptake was therefore 29.9% higher for the suction-cup format. These indirect estimates assume that the loading-solution volume remained unchanged and that extract depletion represented uptake by the specimens.

Release was monitored over 60 min to reflect the proposed application period, providing a short-term comparison of the two hydrogel formats rather than an experimentally established completion time for release. The apparent pomegranate-extract-equivalent concentration in each collected fraction was highest during the first 5 min, reaching 125.4 ± 10.4 µg/mL for the flat hydrogels and 324.9 ± 69.3 µg/mL for the suction-cup hydrogels (Figure 4b). Concentrations subsequently decreased and fluctuated at lower values over the remainder of the experiment. Because the entire 6 mL release medium was renewed at each sampling time, these concentrations describe release during individual 5 min intervals rather than accumulation in a single vessel.

The greater initial loading provides one explanation for the higher cumulative release from the suction-cup hydrogels. At 60 min, the cumulative apparent pomegranate-extract-equivalent amounts released were 4.208 ± 0.354 mg for the flat hydrogels and 6.976 ± 0.681 mg for the suction-cup hydrogels (mean ± SD, *n* = 3). The cumulative amount released by 60 min was therefore 65.8% higher for the suction-cup hydrogels. Relative to the estimated initial loading, these amounts corresponded to approximately 15.2% and 17.6%, respectively (Figure 4c). The percentage curves were calculated by dividing each specimen’s cumulative released amount by the corresponding group loading estimate and multiplying by 100. Their means are therefore equivalent to ratios of group means, rather than percentages based on specimen-matched loading measurements. The error bars reflect variability among the three release specimens and exclude uncertainty in the loading estimates. The results suggest that the higher absolute release from the suction-cup format reflects both greater initial loading and a modestly higher estimated fraction released over the measurement period. Surface-associated extract may contribute to early transfer [23], while extract solution retained within the cup cavities during transfer to the release medium may also contribute to the measured response.

The differences in uptake and release are consistent with the fabricated architecture affecting liquid access and transport. Earlier patterned-hydrogel studies have also examined effects on uptake and release [17,24]. However, external surface area, local thickness, and diffusion paths changed together in the present specimens, so their individual contributions could not be separated. The comparison therefore describes uptake and release from the complete fabricated format under the stated conditions. It does not establish that surface area alone controls release or demonstrate improved directional delivery to skin.

The release profiles were analyzed using cumulative extract-equivalent mass calculated from the successive 6 mL collections, and the R^2^ values for the five kinetic models are compared in Figure 4d. For the flat hydrogels, the zero-order model gave R^2^ = 0.9071, while the empirical power-law model provided a closer description of the profile (R^2^ = 0.9972). For the suction-cup hydrogels, the origin-constrained Higuchi model described the profile well (R^2^ = 0.9962), although the empirical power-law and Weibull models gave slightly higher R^2^ values of 0.9973 and 0.9974, respectively. Model equations, fitted parameters, uncertainty estimates, and residual errors are provided in the Supporting Information. These small differences in fit quality do not establish a unique release mechanism. The Higuchi relationship [25,26] is interpreted here as a description of the observed time dependence rather than proof of exclusive diffusion control. Although initial loading was estimated, the total releasable amount was not established, and neither cumulative profile reached a clear plateau within 60 min. Fitted limiting release amounts therefore remain uncertain.

### 2.5. Cytocompatibility of the Pomegranate-Extract-Containing Hydrogels

HaCaT keratinocytes maintained high metabolic viability across the complete dilution series (Figure 5a). Mean viability remained at or above 96.62% from 0 to 50% extract and was 90.08 ± 1.94% after exposure to the undiluted 100% extract. Thus, all HaCaT group means remained well above the 70% non-cytotoxicity criterion. HDFs showed a stronger concentration-dependent response (Figure 5b). Mean viability ranged from 87.75 to 94.36% at extract concentrations of 1.56–25%, before decreasing to 78.91 ± 0.78% at 50% and 77.79 ± 3.18% at 100%. Although the fibroblast response was lower than the keratinocyte response at the highest concentrations, every HDF group mean remained above the 70% criterion. Representative source micrographs were consistent with a concentration-dependent cellular response (Figure 5a,b). HaCaT cells formed a dense cobblestone-like monolayer in the control and 25% conditions, whereas the 100% extract field showed reduced monolayer uniformity and altered cell appearance. HDFs retained an elongated, spindle-like phenotype, with visibly lower cell density at 100% extract. These observations are qualitative because morphology and confluence were not quantified, and scale information was not available in the supplied source images. Taken together, the MTT data indicate that the selected extract-containing hydrogel was cytocompatible with both cell types under the tested 24 h indirect-extract conditions. The results support the absence of an in vitro cytotoxic response according to the stated 70% criterion; they should not be interpreted as demonstrating complete product or clinical safety.

## 3. Conclusions

The formulation-matched comparison showed that converting a flat NVP/HEMA/CMC hydrogel into a suction-cup format altered early hydration, apparent extract uptake, and short-term release. Relative to the flat hydrogel, the suction-cup format had a 21.4% higher nominal external surface-area-to-dry-mass ratio and a 23.1% greater short-term swelling response, while equilibrium water contents remained comparable. Estimated pomegranate-extract uptake per gram of hydrated hydrogel was 29.9% higher, and the cumulative apparent extract-equivalent amount released over 60 min was 65.8% higher. Release corresponded to approximately 15.2% and 17.6% of the estimated initial loading for the flat and suction-cup hydrogels, respectively. These findings suggest that the higher absolute release from the suction-cup format reflects both greater initial loading and a modestly higher estimated fraction released, although loading was determined indirectly from solution depletion.

The hydration results are consistent with increased surface area facilitating water uptake. However, surface area, local thickness, liquid retention within the cups, and diffusion paths changed together, so their individual contributions could not be separated. The kinetic fits describe the observed release profiles but do not establish a unique mechanism or demonstrate exclusive diffusion control. The bulk-release experiment also did not reproduce directional transfer to skin. Within these limits, suction-cup patterning provides a means of adjusting short-term hydrogel hydration and botanical-extract uptake and release without changing the formulation. Further validation using compound-specific analysis and release or deposition testing on a relevant skin model is needed to determine whether these differences improve performance in cosmetic or biomedical applications.

## 4. Materials and Methods

### 4.1. Materials

2-Hydroxyethyl methacrylate (HEMA), N-vinyl-2-pyrrolidone (NVP), poly(ethylene glycol) diacrylate (PEGDA; nominal number-average molecular mass, 575 g mol^−1^), and 2-hydroxy-2-methylpropiophenone were obtained from Sigma-Aldrich (St. Louis, MO, USA). Carboxymethyl cellulose (CMC) was supplied by LANA GROW CHEMICAL Co., Ltd. (Samut Sakhon, Thailand), and aqueous pomegranate peel extract (2% *w*/*w*) was obtained from the Phytochemical Research Centre (Chiang Mai, Thailand). All reagents were used as received.

For the cytocompatibility assay, thiazolyl blue tetrazolium bromide (MTT) was obtained from Sigma-Aldrich and analytical-grade dimethyl sulfoxide (DMSO) from RCI Labscan (Bangkok, Thailand). HaCaT human keratinocytes were obtained from Elabscience Biotechnology Inc. (Cat. No. CL-0090; Houston, TX, USA). Primary human dermal fibroblasts (HDFs) were isolated from neonatal circumcision foreskin tissue under a protocol approved by the Naresuan University Institutional Review Board (Approval No. 0140/62). High-glucose Dulbecco’s modified Eagle medium (DMEM), fetal bovine serum (FBS), penicillin–streptomycin, and 0.25% trypsin–EDTA were obtained from Gibco (Thermo Fisher Scientific, Waltham, MA, USA).

### 4.2. Mold Fabrication and Suction-Cup Geometry

Three-dimensional printed master molds containing the inverse suction-cup pattern were used to prepare silicone working molds. The silicone elastomer base and curing agent were mixed, degassed under vacuum, and poured over the master molds. After curing for 24 h at room temperature, the silicone molds were separated from the masters and used to cast the hydrogel precursor. The structured mold contained suction cups with an upper outer diameter of 4.0 mm, a lower outer diameter of 2.5 mm, a height of 1.0 mm, and a wall thickness of 0.4 mm. Adjacent cups were separated by a center-to-center distance of 5.0 mm. A planar mold was used to prepare the corresponding flat hydrogel control.

### 4.3. Precursor Preparation and Hydrogel Fabrication

The hydrogel precursor contained 1.000 g NVP, 2.000 g HEMA, 0.120 g PEGDA 575, 0.003 g CMC, and 0.100 g 2-hydroxy-2-methylpropiophenone. NVP, HEMA, PEGDA, and CMC were mixed for 1 h. The photoinitiator was then added, and mixing was continued for a further 1 h. The precursor was cast into the flat and suction-cup molds, and both geometries were photopolymerized under UV irradiation from a 395 nm UV-LED lamp operating at a power of 50 W (220 V AC) positioned at a distance of 7.5 cm for 60 min. After polymerization, the hydrogels were removed from the molds and soaked in distilled water for 24 h, changing the water three times to remove unreacted monomers before testing.

### 4.4. Equilibrium Water Content

The percentage of equilibrium water content (%EWC) was determined using a gravimetric technique. The swollen hydrogel sheets (W_s_) were weighed and then dehydrated in an oven at 60 °C for 72 h. Then, the dehydrated hydrogel sheets (W_d_) were weighed. The %EWC was reported until the weight of the samples reached a constant value. Each hydrogel sheet was measured three times under the same conditions, and the average values were reported using the following equation [27]:

%EWC = (W_s_ − W_d_)/W_s_) × 100%

where W_s_ and W_d_ are the swollen and dried weights of the hydrogel sheets, respectively.

### 4.5. Swelling Test

The swelling behavior of the hydrogel sheets was studied by completely immersing the samples in deionized water at room temperature. The swollen hydrogel sheets were taken out of the deionized water, excess water at the surface was removed, and the sheets were then weighed at each time interval, ranging from 5 to 120 min. The % swelling was calculated based on the change in weight using the following equation [28]:

$$\%swelling=\frac{W_{f}-W_{i}}{W_{i}}\times 100\%$$

where W_i_ and W_f_ are the initial weight and final weight at different times, respectively. The samples were measured three times for each sample and reported as the average % swelling with the standard deviation.

### 4.6. Mechanical Testing

#### 4.6.1. Uniaxial Tensile Testing

Uniaxial tensile tests were performed using a universal testing machine (Instron 5965, Instron Corporation, Norwood, MA, USA) equipped with a 100 N load following a procedure adapted from ASTM D638. Flat and suction-cup hydrogels were cut into strips 10 mm wide. The thickness of each specimen was measured before testing and used with the specimen width to calculate its initial cross-sectional area. Specimens were mounted in the testing machine with a gauge length of 30 mm. Samples were extended until failure at a speed of 30 mm min^−1^. Stress was calculated by dividing the measured force by the initial cross-sectional area. Strain was calculated from the change in grip separation relative to the initial gauge length. Tensile strength, elongation at break, and Young’s modulus were obtained from the resulting stress–strain curves. Young’s modulus was determined from the initial linear region. Results are presented as mean ± SD.

#### 4.6.2. Lap-Shear Testing

Lap-shear tests were performed using a universal testing machine (Instron 5965) with artificial leather fixed to glass slides as the test substrate. Flat and suction-cup hydration hydrogels were placed between two substrate-covered glass slides in an overlapping configuration. Before testing, a 50 g preload was applied for 3 min to establish contact between the hydrogel and the substrate. The assemblies were mounted in the testing machine and extended at 10 mm min^−1^ until interfacial failure.

The maximum force recorded during detachment was used to calculate the apparent lap-shear strength:

τ = F_max_/A_0_

where F_max_ is the maximum force, and A_0_ is the effective initial contact area [29]. For the flat hydrogels, A_0_ was the complete nominal overlap area of 2.5 × 2.5 cm (6.25 cm^2^). For the suction-cup hydrogels, the effective contact area was calculated from the combined annular rim area of the nine cups engaged with the substrate. The contact area of an individual cup was calculated as

A_cup_ = (π/4)(D_o_^2^ − D_i_^2^)

where D_o_ and D_i_ are the measured outer and inner rim diameters, respectively. Results are presented as mean ± SD.

### 4.7. Pomegranate-Extract Loading and Release

Hydrated flat and suction-cup hydrogels were loaded by immersion in 12 mL of a 2% *w*/*w* aqueous pomegranate peel extract solution for 24 h at room temperature (25 °C). Apparent extract uptake was estimated from the decrease in concentration of the loading solution, assuming that the solution volume remained unchanged and that extract depletion represented uptake by the specimens. The concentration difference, expressed in mg/mL, was multiplied by the 12 mL loading volume to estimate the amount incorporated. Mass-normalized uptake was calculated by dividing the estimated uptake amount by the corresponding mean hydrated specimen mass and was expressed as mg/g hydrated hydrogel. After loading, each specimen was transferred to an individual vessel containing 6 mL of DI water as the release medium and vortexed for 1 min before testing. At each 5 min sampling interval over 60 min, the hydrogel specimen was transferred to a new vessel containing 6 mL of fresh DI water. A 200 µL aliquot was taken from each collected medium and analyzed at 290 nm [30] using a Synergy H1 microplate reader (BioTek Instruments, Santa Clara, CA, USA). Release measurements were performed using three independent hydrogel specimens per format, with the same specimens followed throughout the experiment. Apparent pomegranate-extract-equivalent concentrations were determined using a calibration curve created using the same pomegranate peel extract (Figure S1). The calibration relationship was A_290_ = 44.994C − 0.0137, where A_290_ is the blank-corrected absorbance, and C is the extract concentration expressed as a numerical percentage. Concentrations were converted to mg/mL as C = 10(A_290_ + 0.0137)/44.994. Conversion from a mass fraction to a mass-per-volume concentration assumed an aqueous solution density of 1 g/mL. Because the calibration used the complete extract, the reported concentrations represent apparent extract equivalents rather than concentrations of individual constituents.

The concentration in each collected fraction was expressed in µg/mL. The amount released during each interval was calculated by multiplying its concentration in mg/mL by the complete collection volume of 6 mL. These interval amounts were summed separately for each specimen to obtain cumulative release in mg. Cumulative percentage release was calculated as 100 times the cumulative amount divided by the estimated initial loading, using fixed group estimates for both the flat hydrogels and suction-cup hydrogels. Results are reported as mean ± SD across the three release specimens (*n* = 3).

Release kinetics were evaluated by fitting zero-order, first-order, Higuchi [25], empirical power-law, and Weibull models, without additive offsets, to the mean cumulative extract-equivalent mass profiles over 5–60 min. Fits used unweighted bounded least squares on the original mass scale. The first-order model was fitted directly as Q(t) = Q∞[1 − exp(−k_1_t)], with Q∞ estimated as a model parameter rather than fixed to the loading estimate. Fit quality was assessed using the coefficient of determination (R^2^) [26] and root-mean-square error, alongside parameter uncertainty. Model equations, parameter units, fitting bounds, and numerical results are provided in the Supporting Information.

### 4.8. Cytocompatibility of the Pomegranate-Extract-Containing Hydrogels

The cytocompatibility of the selected pomegranate-extract-containing NVP/HEMA/CMC hydrogel was evaluated by an indirect extraction method using HaCaT human keratinocytes and primary human dermal fibroblasts (HDFs). Cells were maintained in DMEM supplemented with 10% FBS and 1% penicillin–streptomycin at 37 °C in a humidified atmosphere containing 5% CO_2_. Hydrogel specimens were fully submerged in culture medium at a surface-area-to-medium ratio of 3 cm^2^ mL^−1^ and extracted for 24 h at 37 °C on an orbital shaker at 60 rpm, following the sample-preparation approach described in ISO 10993-12 [31]. The resulting 100% extract was filtered and serially diluted with fresh culture medium to obtain concentrations of 50, 25, 12.5, 6.25, 3.13, and 1.56% (*v*/*v*). HaCaT and HDF cells were seeded in 96-well plates at 1 × 10^4^ cells per well and allowed to attach for 24 h. The culture medium was then removed, the cells were washed with sterile PBS (pH 7.4), and fresh hydrogel extract was added at the specified concentrations. After a further 24 h of exposure, metabolic activity was measured using the MTT assay. The resulting formazan crystals were dissolved in DMSO, and absorbance was measured at 570 nm using a microplate reader. Cell viability was expressed relative to the corresponding 0% extract control. Results are reported as mean ± SD from triplicate measurements (*n* = 3, individual specimens), as recorded in the source dataset. Mean viability below 70% of the control was interpreted as indicating cytotoxic potential according to ISO 10993-5 [32].

## Figures and Tables

**Figure 1:**
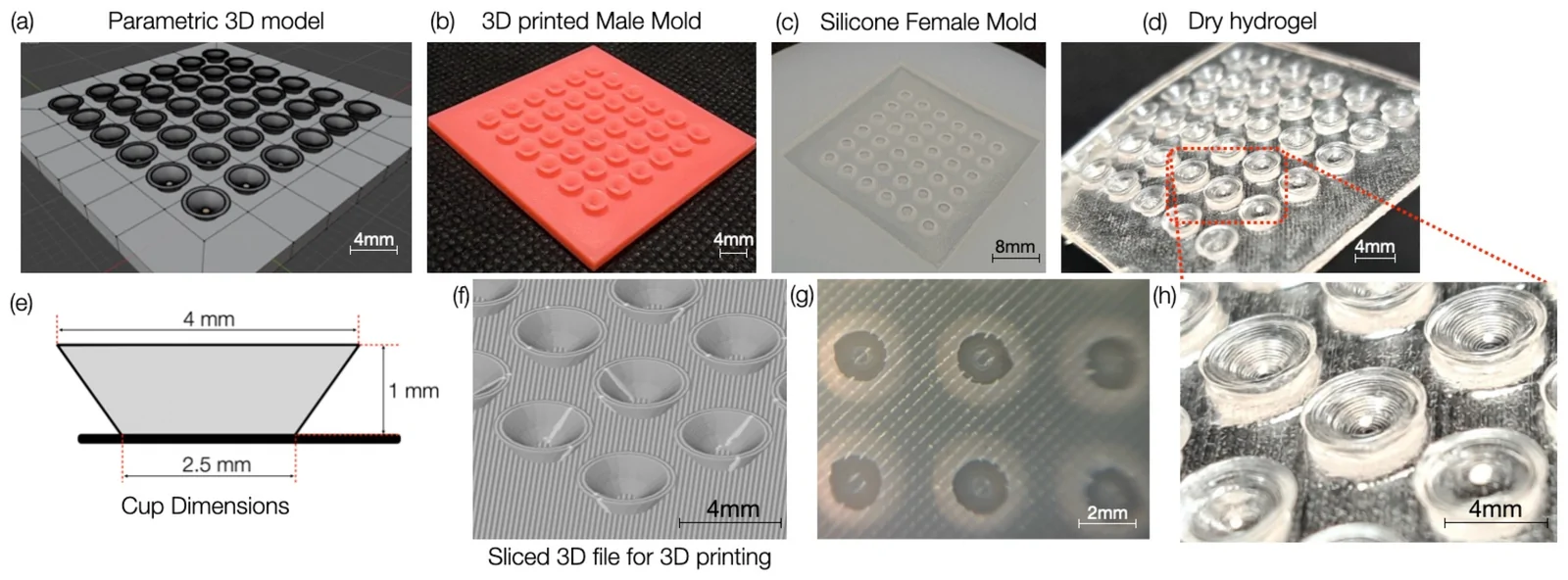
Design and fabrication of the suction-cup hydrogel array: (**a**) Parametric three-dimensional model of the cup array. (**b**) The 3D-printed male master. (**c**) Silicone female mold replicated from the printed master. (**d**) Dry structured hydrogel after photopolymerization and demolding. (**e**) Nominal cross-sectional dimensions of the cup design. (**f**) Sliced print file showing the cup geometry. (**g**) Enlarged view of the replicated mold features. (**h**) Enlarged view of the dry hydrogel showing retention of the cup rims and cavities.

**Figure 2:**
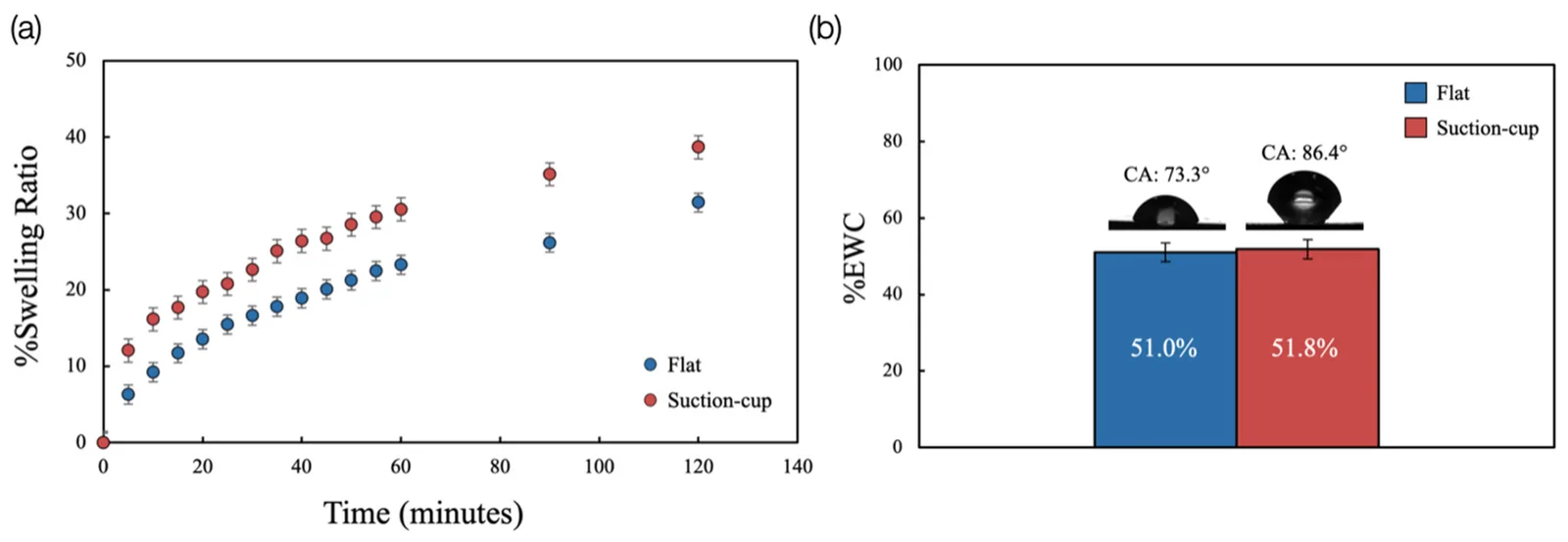
Hydration behavior of flat and suction-cup hydrogels: (**a**) Percentage mass increase during immersion for 120 min. Symbols represent mean values, and error bars represent standard deviations (*n* = 3). (**b**) Equilibrium water content of the flat and suction-cup hydrogels. Bars represent means, and error bars represent standard deviations (*n* = 3). Representative initial water-droplet profiles obtained using 5 µL droplets are shown above the bars.

**Figure 3:**
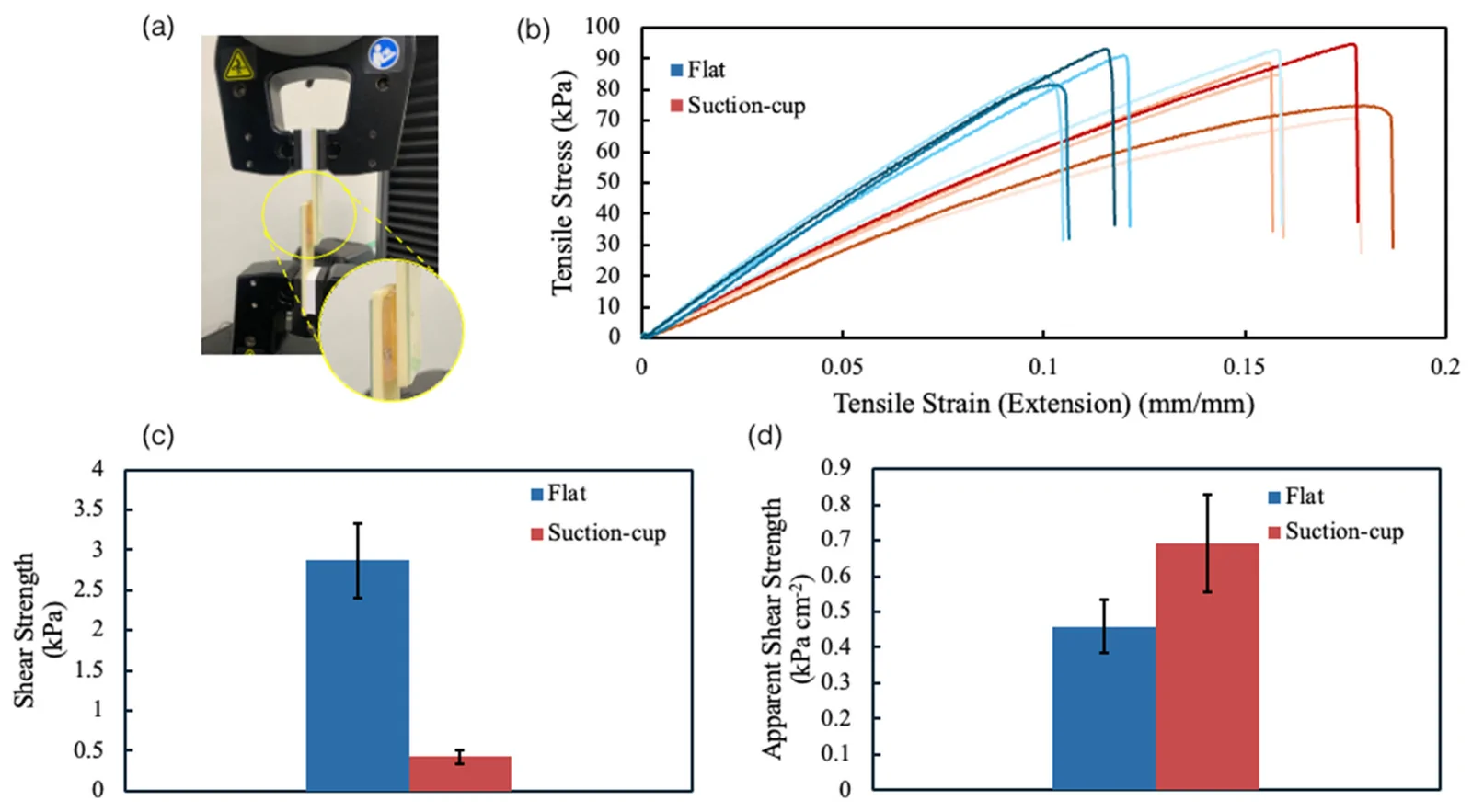
Mechanical response of flat and suction-cup hydrogels: (**a**) Uniaxial tensile-test configuration. (**b**) Individual tensile stress–strain profiles for flat and suction-cup hydrogels (*n* = 5). (**c**) Maximum lap-shear strength of the two hydrogel formats. Error bars show standard deviations. (**d**) Apparent shear strength of flat and suction-cup hydrogels.

**Figure 4:**
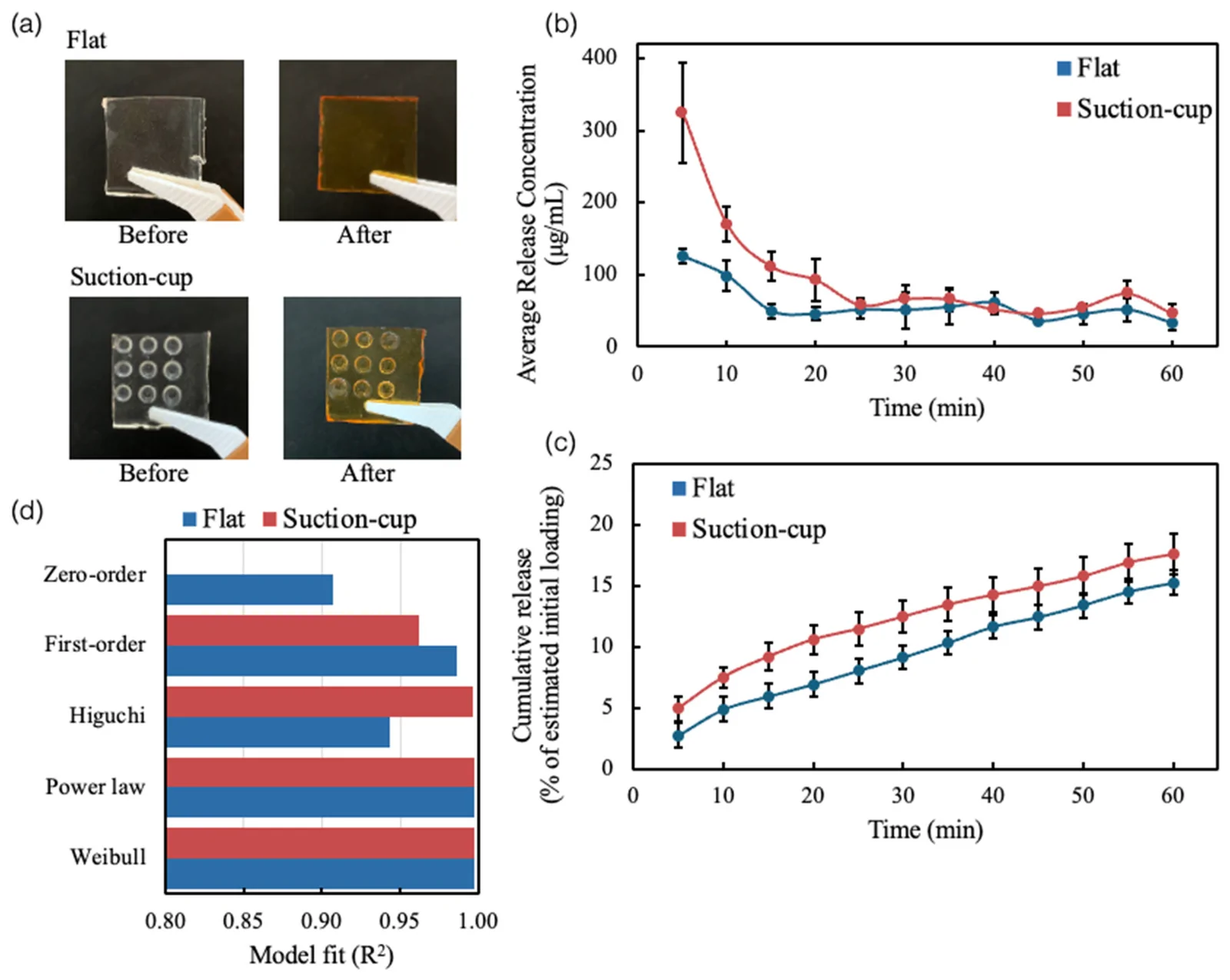
Pomegranate-extract uptake and release from flat and suction-cup hydrogels: (**a**) Hydrogel specimens before and after extract loading. (**b**) Apparent pomegranate-extract-equivalent concentration in successive 6 mL release-medium fractions collected at 5 min intervals. (**c**) Cumulative release expressed as a percentage of estimated initial loading, using group loading estimates of 27.6 mg for flat hydrogels and 39.6 mg for suction-cup hydrogels. Points in (**b**,**c**) represent means ± SD from three independent release specimens. (**d**) Coefficients of determination (R^2^) for zero-order, first-order, Higuchi, empirical power-law, and Weibull models.

**Figure 5:**
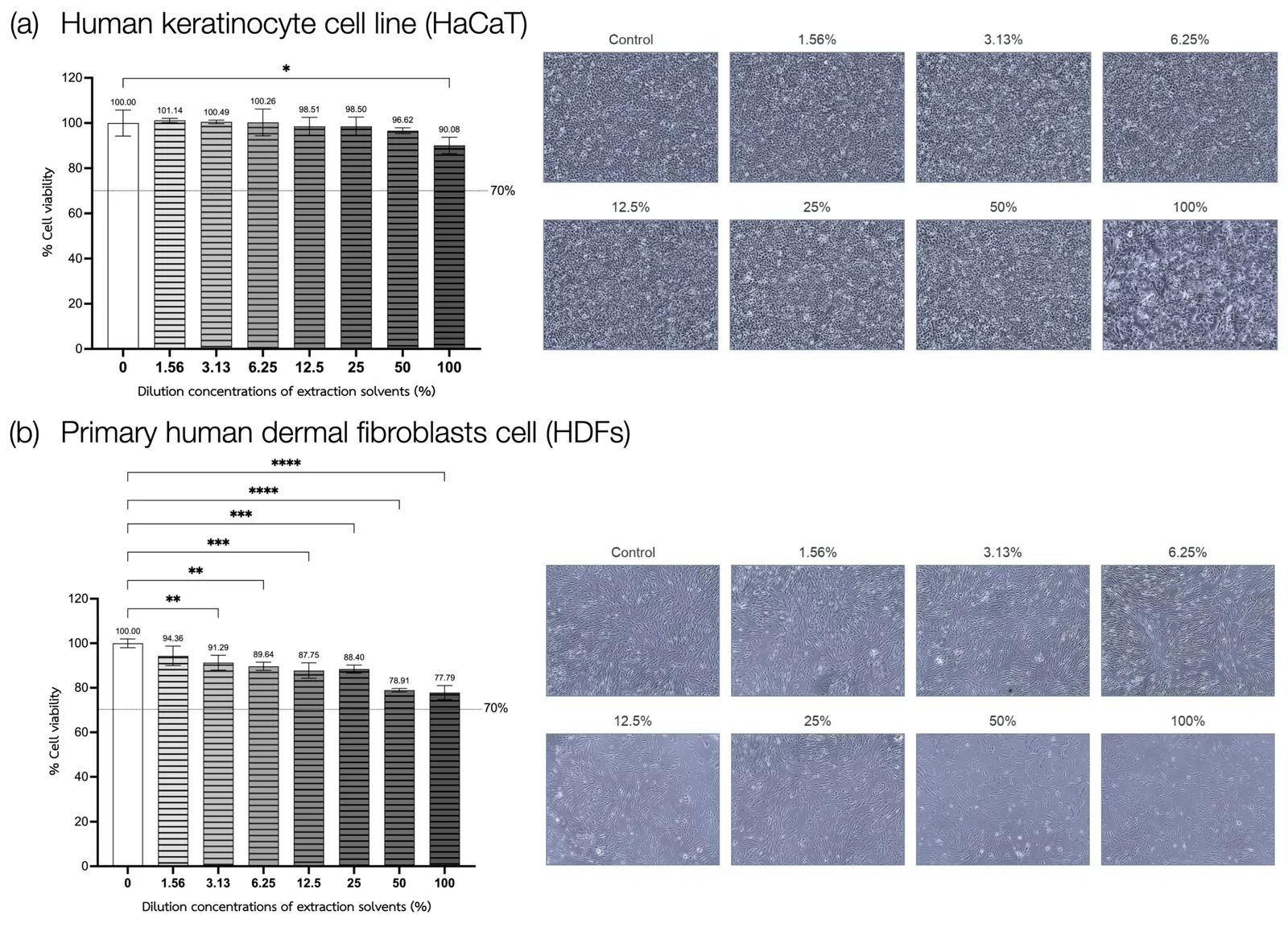
(**a**) Cytocompatibility of the NVP/HEMA/CMC hydrogel with HaCaT keratinocytes and human dermal fibroblasts (HDFs): (**a**) HaCaT cell viability measured by the MTT assay and representative microscopy images after 24 h of exposure to 0–100% (*v*/*v*) hydrogel extracts. (**b**) HDF viability and corresponding microscopy images under the same conditions. Data are presented as mean ± SD (*n* = 3). The dotted horizontal line indicates the 70% viability threshold for a non-cytotoxic response. Statistical significance relative to the control was determined by one-way ANOVA followed by Dunnett’s multiple-comparison test: * *p* < 0.05, ** *p* < 0.01, *** *p* < 0.001, and **** *p* < 0.0001.

## Data Availability

The data supporting the findings of this study are available from the corresponding author upon reasonable request.

## Supplementary Materials

The following are available online at https://www.mdpi.com/article/10.3390/gels12100860/s1, Figure S1: Calibration curve for aqueous pomegranate peel extract at 290 nm. Squares represent the mean blank-corrected absorbance recorded at each of the seven calibration concentrations; the solid line is the linear least-squares fit. Concentration is expressed as a numerical percentage (for example, 0.0625 denotes 0.0625%); Table S1: Loading estimates and cumulative release at 60 min; Table S2: Model equations and parameter units, with t in minutes; Table S3: Parameters fitted to the mean of three flat release profiles, 5–60 min. Parameter values are estimates ± conditional fit SE; units are defined in S3. These are not mean ± SD of specimen-level parameter estimates; Table S4: Parameters fitted to the mean of three suction-cup release profiles, 5–60 min. Parameter values are estimates ± conditional fit SE; units are defined in S3. These are not mean ± SD of specimen-level parameter estimates.
