# Supplementary material for: Bioinspired Suction-Cup Architecture Modulates Hydration and Pomegranate-Extract Release from NVP/HEMA/CMC Hydrogels
Source: Gels. 2026 Sep 24;12(10):860. doi: 10.3390/gels12100860 (PMC13704729; doi:10.3390/gels12100860)
Supplement: Supplementary file 1 [file gels-12-00860-s001.zip › gels-4547033-supplementary.pdf]

Supporting information

S1. Pomegranate extract calibration

The calibration used the same pomegranate peel extract as the loading and release experiments. Figure S1 shows the seven calibration concentrations used for the reported linear relationship, spanning 0.0009766–0.0625%. Blank-corrected absorbance at 290 nm was fitted against the numerical percentage concentration C, giving $A_{290} = 44.994C - 0.0137$ ($R^2 = 0.9993$).

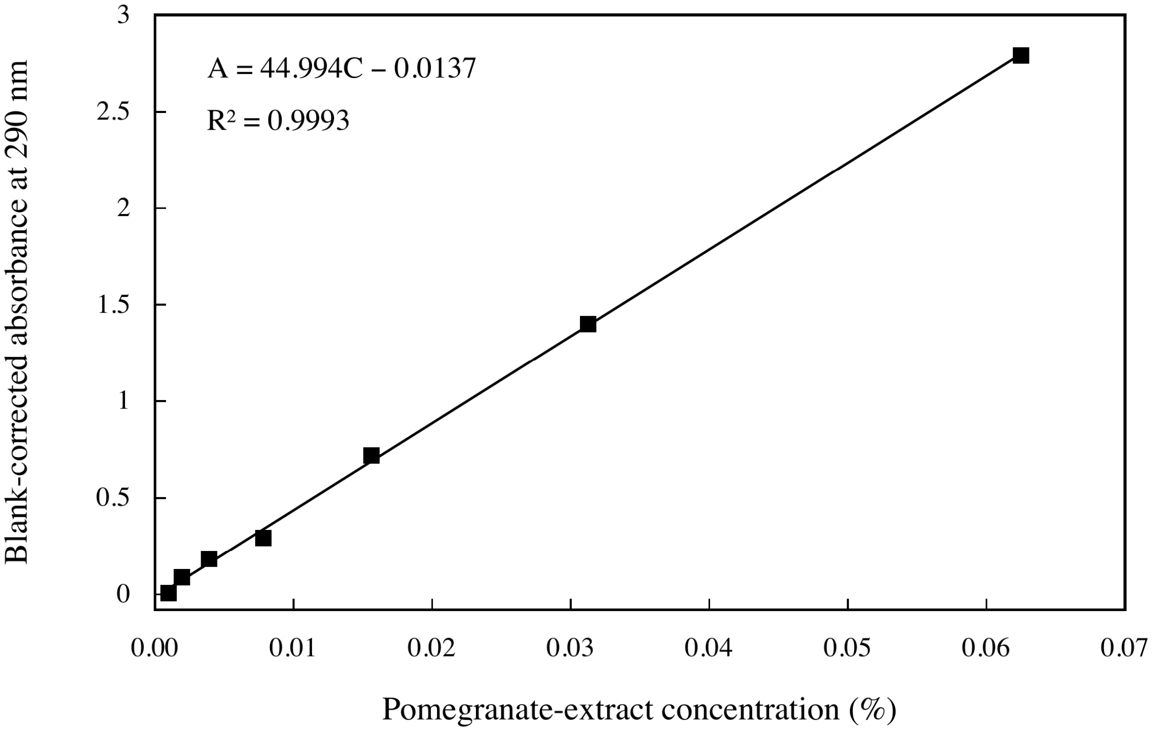

Figure S1. Calibration curve for aqueous pomegranate peel extract at 290 nm. Squares represent the mean blank-corrected absorbance recorded at each of the seven calibration concentrations; the solid line is the linear least-squares fit. Concentration is expressed as a numerical percentage (for example, 0.0625 denotes 0.0625%).

Extract-equivalent concentration was calculated as C (%) = (A_{290} + 0.0137)/44.994. Conversion to mg/mL used C (mg/mL) = 10C (%), assuming a solution density of 1 g/mL for the mass/mass concentration basis. The calibration measures the combined UV response of the complete extract and does not quantify individual constituents. The rounded calibration coefficients above were retained for the release calculations.

S2. Release data and loading normalization

This supplement reports kinetic model definitions, fitted parameters and fit diagnostics for apparent pomegranate-extract-equivalent release from flat and suction-cup hydrogels. Fits describe the measured 5–60 min interval and do not establish an exclusive release mechanism.

Three independent specimens were followed for each format, with 12 measurements per specimen at 5 min intervals. At each collection, the hydrogel was transferred to 6 mL of fresh release medium. A 200 µL aliquot from the collected medium was analysed by UV absorbance. Each concentration therefore describes an individual collection interval.

Concentrations were calculated using the calibration in Figure S1 and converted to mg/mL as described in Section S1. Apparent released mass in each 6 mL collection was $6C_i$, where C_i is the concentration in mg/mL. Cumulative mass Q_n was the sum of successive interval masses within each specimen.

Cumulative percentage release was calculated as $P_n = 100Q_n/L$, with fixed group loading estimates L = 27.6 mg for flat specimens and 39.6 mg for suction-cup specimens. These estimates derive from depletion of a nominally unchanged 12 mL bath from 2.00% to 1.77% and 1.67%, respectively. They assume that bath depletion represents specimen uptake. They are not individually matched loading measurements, and no loading replicate count or loading SD is assigned here.

Table S1. Loading estimates and cumulative release at 60 min.

Format	Loading estimate (mg)	Q at 60 min (mg)	P at 60 min (%)
Flat	27.6	4.208 ± 0.354	15.25 ± 1.28
Suction-cup	39.6	6.976 ± 0.681	17.62 ± 1.72

Values for Q are mean ± sample SD across the three release specimens. For P, the SD is rescaled using the fixed group denominator and excludes uncertainty in loading. The denominator represents estimated initial loading, not an experimentally established total releasable amount.

Multiplication of the response by a constant leaves R^2 and fitted shape parameters unchanged when the model and parameter constraints are scaled consistently. Amount-based parameters and RMSE change in proportion to the scale factor. The normalisation does not supply an additional measurement or establish a release plateau.

S3. Model definitions and fitting methods

Table S2. Model equations and parameter units, with t in minutes.

Model	Cumulative mass equation	Parameter units
Zero-order	$Q(t) = k_0t$	k_0: mg/min
Higuchi	$Q(t) = kH\sqrt{t}$	kH: mg/min½
First-order	$Q(t) = Q\infty[1 - \exp(-k_1t)]$	$Q\infty$: mg; k_1: min^{-1}
Empirical power law	$Q(t) = at^n$	a: mg/min^n; n: dimensionless
Empirical Weibull	$Q(t) = Q\infty\{1 - \exp[-(t/\tau)^\beta]\}$	$Q\infty$: mg; τ: min; β: dimensionless

All five models were fitted without additive offsets using unweighted bounded least squares on the original cumulative mass scale, with multiple initial guesses for nonlinear asymptotic models. No logarithmic response transformation was applied, and no zero-time observation was added. The tables below report fits to the mean cumulative release profiles.

Cumulative ordinary least squares (OLS) minimised $\Sigma[Qobserved(t) - Qfitted(t)]^2$.

$R^2 = 1 - SSEcumulative/SSTcumulative$ uses the centred cumulative response for every model. RMSE $= \sqrt{(SSE/12)}$. Parameter SE values are local Jacobian estimates using the residual variance SSE/(12 − p), where p is the number of fitted model parameters. Conditional 95% intervals can be calculated as estimate ± $t_{0.975,12-p}$ × SE. These estimates exclude calibration and loading uncertainty. Cumulative-fit SEs are descriptive because cumulative errors are correlated.

The empirical power-law exponent is not assigned a Korsmeyer–Peppas mechanism category. A fractional loading denominator alone does not validate the total-releasable fraction range or the geometry assumptions needed for such an interpretation. Free limiting amounts are fitted quantities, not measured loads.

S4. Mean cumulative release fits for flat hydrogels

Table S3. Parameters fitted to the mean of three flat release profiles, 5–60 min. Parameter values are estimates ± conditional fit SE; units are defined in S3. These are not mean ± SD of specimen-level parameter estimates.

Model	Parameters ± conditional SE	R^2	RMSE (mg)
Zero-order	k0 = 0.07708 ± 0.002645	0.9071	0.3228
Higuchi	kH = 0.4988 ± 0.01334	0.9432	0.2523
First-order	Qinf = 5.929 ± 0.54<br>k1 = 0.01977 ± 0.002815	0.9862	0.1242
Power law (empirical)	a = 0.2444 ± 0.01367<br>n = 0.6949 ± 0.01496	0.9972	0.0558
Weibull (empirical)	$Q\infty$ and τ not identifiable<br>$\beta \approx 0.6949$ (limiting empirical shape)	0.9972	0.0558

For ill-conditioned Weibull fits, limiting amounts and time scales are not interpretable and their local SEs are omitted. An approximately fitted power-law limiting shape may remain identifiable without an identifiable release plateau. Estimates of $Q\infty$ from all models must not be substituted for experimentally measured loading.

The fitting bounds were: k_0 0–10 mg/min; kH 0–20 mg/min½; $Q\infty$ 10^{-8}–10^{7} mg; k_1 10^{-9}–10 min^{-1}; power-law coefficient a 10^{-8}–100 mg/min^n and exponent n 0.01–2; Weibull τ 0.001–10^{10} min and β 0.05–3. Multiple starting points were used to check nonlinear solutions. The flat cumulative Weibull fit reached the τ upper bound.

The empirical power law describes the flat cumulative release profile more closely ($R^2 = 0.9972$) than the zero-order model ($R^2 = 0.9071$). The Weibull model gives the same R^2 to four decimal places, but its limiting amount and time scale are not identifiable. These fits do not establish a unique release mechanism.

S5. Mean cumulative release fits for suction-cup hydrogels

Table S4. Parameters fitted to the mean of three suction-cup release profiles, 5–60 min. Parameter values are estimates ± conditional fit SE; units are defined in S3. These are not mean ± SD of specimen-level parameter estimates.

Model	Parameters ± conditional SE	R^2	RMSE (mg)
Zero-order	k0 = 0.1365 ± 0.008745	0.4713	1.0673
Higuchi	kH = 0.9009 ± 0.00477	0.9962	0.0902
First-order	Qinf = 7.009 ± 0.3105<br>k1 = 0.04566 ± 0.0051	0.9618	0.2870
Power law (empirical)	a = 0.967 ± 0.03364<br>n = 0.4805 ± 0.009499	0.9973	0.0758
Weibull (empirical)	Qinf = 39.32 ± 60.69<br>tau = 1491 ± 5451<br>beta = 0.5124 ± 0.05602	0.9974	0.0744

The suction-cup Weibull fit has large uncertainty in Q_∞ and τ, reflecting the absence of an established release plateau. These estimates should not be interpreted as a measured loading amount or a reliable long-term release prediction.

The fitting bounds were: k_0 0–10 mg/min; kH 0–20 mg/min½; Q_∞ 10^{-8}–10^7 mg; k_1 10^{-9}–10 min^{-1}; power-law coefficient a 10^{-8}–100 mg/min^n and exponent n 0.01–2; Weibull τ 0.001–10^{10} min and β 0.05–3. Multiple starting points were used to check nonlinear solutions.

The origin-constrained Higuchi model describes the suction-cup cumulative release profile well (R^2 = 0.9962). The empirical power-law and Weibull models give slightly higher R^2 values of 0.9973 and 0.9974, respectively. These small differences do not establish a unique mechanism, and the square-root-of-time relationship does not demonstrate exclusive diffusion control.
